# Anesthesia‐free transoral endoscopy using a novel modified pacifier in early infancy

**DOI:** 10.1002/jpn3.70463

**Published:** 2026-06-05

**Authors:** Jonathan A. Berken, Zoe Rosoff‐Verbit, Tyler Babinski, Matthew J. Ryan, the Transpacifier Endoscopy Research Group, Elizabeth Silvestro, Michael A. Manfredi

**Affiliations:** ^1^ Division of Gastroenterology, Hepatology, and Nutrition, Department of Pediatrics Perelman School of Medicine at the University of Pennsylvania and Children's Hospital of Philadelphia Philadelphia Pennsylvania USA; ^2^ Division of Neonatology, Department of Pediatrics Perelman School of Medicine at the University of Pennsylvania and Children's Hospital of Philadelphia Philadelphia Pennsylvania USA; ^3^ Department of Radiology Perelman School of Medicine at the University of Pennsylvania and Children's Hospital of Philadelphia Philadelphia PA USA

**Keywords:** 3D printing, gastrointestinal diagnostics, infant esophagogastroduodenoscopy, pilot study, unsedated

Esophagogastroduodenoscopy (EGD) is the gold standard for evaluating upper gastrointestinal (GI) disorders in infants, with indications including feeding intolerance, suspected inflammatory or structural disease, and post‐surgical surveillance.^1^ Mucosal biopsy obtained during EGD is often essential for diagnosis, particularly for eosinophilic esophagitis (EoE) or chronic diarrhea when upper GI pathology is suspected and more common etiologies have been excluded.^1^ Despite its diagnostic value, endoscopy in neonates and young infants is performed far less frequently than in older children, reflecting differences in indications, diagnostic yield, and procedural risk.^1^

EGD in young infants typically requires general anesthesia, which has been associated with potential adverse neurodevelopmental effects.^2^, ^3^ Experimental and clinical studies have shown that anesthetic exposure during critical developmental periods induces neuroapoptosis, altered synaptogenesis, and subsequent cognitive and behavioral changes.^3^ Although brief, single exposures have not consistently demonstrated measurable harm,^4^ these results nevertheless fuel parental worry in as many as 40% of families and can lead to delays in elective procedures.^2^ While unsedated transnasal endoscopy (TNE) is feasible in older children,^5^ anatomical limitations make it impractical in infants, leaving this population without a non‐anesthetized option.

To address this gap in care, we developed transpacifier endoscopy (TPE), a novel unsedated technique that leverages the infant suck reflex to perform upper GI evaluation. Using a custom 3D‐printed pacifier designed for an ultrathin <3.5 mm endoscope with a 1.7–2.0 mm biopsy channel, TPE allows direct visualization and tissue sampling without anesthesia. This approach eliminates anesthesia‐related neurodevelopmental risks, simplifies procedural logistics, and enables point‐of‐care evaluation. As a result, TPE provides a minimally invasive and physiologically congruent endoscopic methodology in infants.

This prospective, single‐center feasibility study was conducted at the Children's Hospital of Philadelphia (CHOP), a large tertiary pediatric hospital, and was approved by the CHOP Institutional Review Board (IRB #23‐021838). Enrollment occurred between October 2024 and May 2025. Written informed consent was obtained from the parents or legal guardians of all participants. Infants were recruited from inpatient consultations and outpatient visits for feeding difficulties, failure to thrive, and suspected upper GI disorders. Eligible infants were ≤6 months of age (term‐corrected if <36 weeks’ gestation), under the care of a hospital gastroenterologist, and demonstrated an intact suck reflex. Exclusion criteria included hemodynamic instability, baseline mechanical ventilation, suspected upper GI bleeding, cardiac disease, or neurological conditions that would preclude unsedated procedures.

Design and fabrication of the modified pacifier used for TPE was carried out in collaboration with CHOP's 3D printing laboratory. Figure S1 shows the computer‑aided design, featuring a central aperture for passage of the ultrathin endoscope, concave platform to stabilize the endoscope, and lateral flanges to secure the fixation strap. Pacifiers were modeled in SolidWorks (Dassault Systemes) and produced on a Formlabs 3B printer using BioMed Flex 80A resin (Formlabs). All devices were manufactured in accordance with institutional guidelines and discarded after single use.

TPE was performed in the Endoscopy Suite rather than the operating room, where infants at our institution typically undergo endoscopy under general anesthesia. Infants were kept *nil per os* for 2 h before the procedure. The pacifier was dipped in Sweet‐Ease sucrose solution to facilitate acceptance, with additional sucrose administered through the pacifier openings as needed during the procedure. The Olympus BF‐XP190 endoscope was advanced through the central aperture to visualize the esophagus and stomach, and biopsies were obtained. Infants were comforted by a caregiver or nurse throughout and continuously monitored for heart rate, oxygen saturation, blood pressure, and respiratory rate. After TPE, infants were allowed to resume feeding or use a pacifier and were observed for 5–10 min before discharge. Adverse events, including aspiration, post‐biopsy bleeding, procedural distress, gagging, airway obstruction, and skin irritation, were monitored.

Caregivers completed a satisfaction survey following their infant's TPE procedure. This five‐item REDCap survey assessed their understanding of anesthesia avoidance, procedural anxiety, infant comfort, satisfaction, and willingness to repeat TPE.

Pediatric gastroenterologists at CHOP were invited to complete an anonymous 6‐item REDCap survey assessing perceptions of TPE feasibility, clinical utility, advantages over anesthesia‐based EGD, and willingness to adopt the technique.

Primary outcomes were feasibility (completion without anesthesia) and safety (hemodynamic stability and adverse events). Secondary outcomes included biopsy adequacy for histologic evaluation, assessed by a pathologist blinded to the study objectives, along with parent‐reported satisfaction and physician attitudes. All data were recorded in REDCap and summarized descriptively. Feasibility was calculated as the proportion of completed procedures, biopsy adequacy as tissue suitable for histology, and adverse events were categorized by type and severity. Survey responses were reported as frequencies, percentages, and distributions across the 5‐point Likert scale.

Six infants, aged 2–6 months (mean 4.5 months), successfully underwent TPE. Each procedure was completed in full in under 10 min, achieving adequate visualization of the esophagus and stomach (Table 1). The modified pacifier functioned as intended, allowing passage of the endoscope, and 2–4 biopsies were obtained in all cases. All procedures were performed under standard clinical monitoring, and vital signs (heart rate, blood pressure, and oxygen saturation) indicated hemodynamic stability throughout each procedure. No major adverse events occurred; minor events such as transient gagging and small‐volume spit‐up were self‐limited and did not interrupt the procedures. Within 15 min, all infants resumed oral feeding or returned to their usual pacifier. Endoscopic visualization and diagnostic yield were comparable to conventional sedated EGD (Figure 1A,C). Histology was normal or non‐diagnostic in most cases; two infants (Patients 4 and 6) showed rare intraepithelial eosinophils not meeting criteria for EoE (Figure 1B,D). TPE led to changes in therapy in two of the six patients.

**Table 1:** Characteristics and clinical outcomes of study subjects (*n* = 6).

Subject	Age (months)	Sex	Indication	Biopsy site	Visual findings	Pathologic findings	Adverse events	Outcomes
1	3.5	Female	Reflux	Esophagus, stomach	Normal esophagus/stomach	Normal	None	Medication change
2	2	Male	Feeding difficulty, vomiting	Esophagus	Normal esophagus/stomach	Normal	Gagging	—
3	5.5	Male	Formula intolerance	Esophagus, stomach	Normal esophagus/stomach	Normal	Spit‐up	—
4	6	Male	Reflux	Esophagus, stomach	Normal esophagus/stomach	Squamous mucosa, rare eosinophils (0–3/hpf); oxyntic gastric mucosa	None	—
5	5	Male	Dysphagia, reflux	Esophagus, stomach	Normal mucosa, copious secretions	Normal	None	Medication change
6	5	Male	Failure to thrive, feeding difficulty	Esophagus, stomach	Normal esophagus/stomach	Squamous mucosa, rare eosinophils (0–2/hpf)	None	—

Abbreviation: hpf, high‐power field.

**Figure 1:**
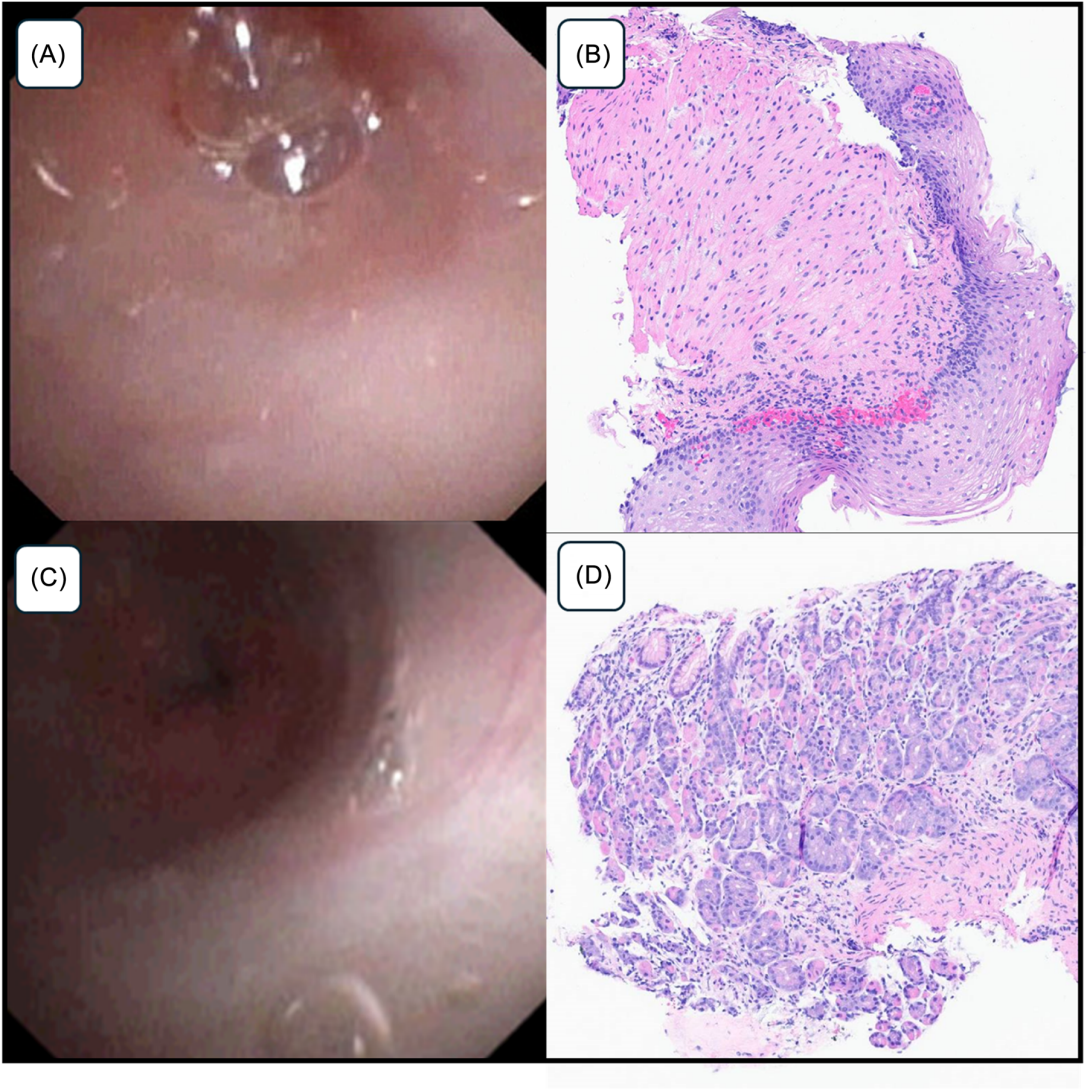
Representative endoscopic and histologic findings obtained using transpacifier endoscopy in a single patient. (A) Endoscopic visualization of the distal esophagus and (B) corresponding histology from esophageal biopsy demonstrating preserved mucosal architecture, both of sufficient quality for pathologic interpretation. (C) Endoscopic view of the prepyloric region of the stomach and (D) histology from the gastric biopsy, also of adequate diagnostic quality. All biopsy specimens obtained through transpacifier endoscopy were deemed of appropriate quality for histopathologic evaluation.

Caregivers reported highly favorable impressions of TPE: all understood anesthesia avoidance and strongly agreed that they were glad their child underwent TPE. Two parents reported pre‐procedure anxiety, but five of six strongly preferred this approach for future procedures. Among pediatric gastroenterologists (*n* = 24), 79% expressed strong interest in adopting TPE, and over 85% agreed that performing endoscopy outside the operating room is a major advantage. Responses from both caregivers and physicians are summarized in Table S1, highlighting the perceived benefits of anesthesia avoidance.

Our early experience demonstrates that unsedated TPE is feasible and safe in infants ≤6 months. All procedures were completed with adequate visualization and diagnostic‐quality biopsies, and findings altered management in two cases. No major adverse events occurred, and parent and provider feedback supported TPE as a viable anesthesia‐free alternative.

TPE offers a critical advantage by avoiding anesthesia during a sensitive neurodevelopmental period. While brief exposures may be low risk,^4^ repeated or prolonged anesthesia remains a concern, particularly for serial evaluations of EoE and post‐operative surveillance of tracheoesophageal fistula/esophageal atresia.^2^, ^3^ Avoiding anesthesia also reduces airway risk, procedural time, and recovery delays.^5^ Cost savings are likely substantial; prior work reports a 53.4% reduction with TNE, potentially greater for infant procedures requiring intensive anesthesia.^6^ TPE has broad utility in infants with persistent feeding difficulties, suspected allergic disease, congenital anomalies, post‐surgical strictures, or unexplained mucosal pathology. By reducing anesthesia burden, it enables longitudinal surveillance and may influence management. Its portable setup also allows bedside and office‐based procedures.

Caregiver and physician feedback was positive, although referral patterns remained conservative, reflecting longstanding barriers to infant endoscopy, including perceived risk, limited indications, anesthesia concerns, and technical challenges.^1^ This hesitancy may contribute to diagnostic blind spots in early infancy, where empiric management often substitutes for mucosal evaluation. By lowering the procedural threshold, TPE may enable earlier identification of treatable conditions and facilitate biopsy‐driven management. Future study participants will undergo TPE using a longer endoscope to enable duodenal access and sampling when indicated, thereby expanding its potential clinical applications in infants. Broader adoption may also clarify the timing and phenotype of EoE onset in infancy and refine early diagnostic frameworks. Further validation in multicenter studies is warranted to assess safety, diagnostic yield, cost‐effectiveness, and reproducibility, alongside standardization of device design, sampling, training, and patient selection.

Overall, TPE offers a novel, anesthesia‐free approach to upper GI evaluation in early infancy, combining a familiar pacifier interface with ultrathin endoscopy to enable safe, point‐of‐care diagnostics. Initial experience demonstrates high procedural success, strong acceptance, and no major adverse events, supporting readiness for broader validation. By preserving diagnostic yield while eliminating anesthesia exposure, TPE has the potential to transform early‐life GI evaluation, expand access, and support longitudinal monitoring in conditions requiring repeated assessment.

## CONFLICT OF INTEREST STATEMENT

The authors declare no conflicts of interest.

## ETHICS STATEMENT

The study was conducted according to the Declaration of Helsinki and its later amendments. The study protocol was approved by the CHOP Ethics Committee. Written informed consent was obtained for each participant by parents/legal guardians.

## Supporting information

Supplemental Figure 1. Design and fabrication of the modified 3D‐printed pacifier used for TPE, highlighting the central aperture for ultrathin endoscope passage, the concave platform for scope stabilization, and lateral flanges for secure strap fixation. Shown the computer‐aided design in the drawing (A) with two standard views (B and C) and the final print (D).

TPE_Supplemental Table 1_20260314.

## References

[1] Mezoff EA , Williams KC , Erdman SH . Gastrointestinal endoscopy in the neonate. Clin Perinatol. 2020;47:413‐422. 10.1016/j.clp.2020.02.012 32439120

[2] Koh JH , Daniel P , Bong CL . Parental perception on the effects of early exposure to anaesthesia on neurodevelopment. Anaesthesia. 2019;74:51‐56. 10.1111/anae.14465 30383296

[3] Davidson A , Flick RP . Neurodevelopmental implications of the use of sedation and analgesia in neonates. Clin Perinatol. 2013;40:559‐573. 10.1016/j.clp.2013.05.009 23972757

[4] Sun LS , Li G , Miller TLK , et al. Association between a single general anesthesia exposure before age 36 months and neurocognitive outcomes in later childhood. JAMA. 2016;315:2312‐2320. 10.1001/jama.2016.6967 27272582 PMC5316422

[5] Friedlander JA , DeBoer EM , Soden JS , et al. Unsedated transnasal esophagoscopy for monitoring therapy in pediatric eosinophilic esophagitis. Gastrointest Endosc. 2016;83:299‐306.e1. 10.1016/j.gie.2015.05.044 26142551 PMC4698253

[6] Nguyen N , Mark J , Furuta GT . Emerging role of transnasal endoscopy in children and adults. Clin Gastroenterol Hepatol. 2022;20:501‐504. 10.1016/j.cgh.2021.11.021 34921762

