# Supplementary material for: Anesthesia‐free transoral endoscopy using a novel modified pacifier in early infancy
Source: J Pediatr Gastroenterol Nutr. 2026 Jun 5;83(2):331–4. doi: 10.1002/jpn3.70463 (PMC13446647; doi:10.1002/jpn3.70463)
Supplement: Supplementary file 1 [file JPN3-83-331-s002.docx]

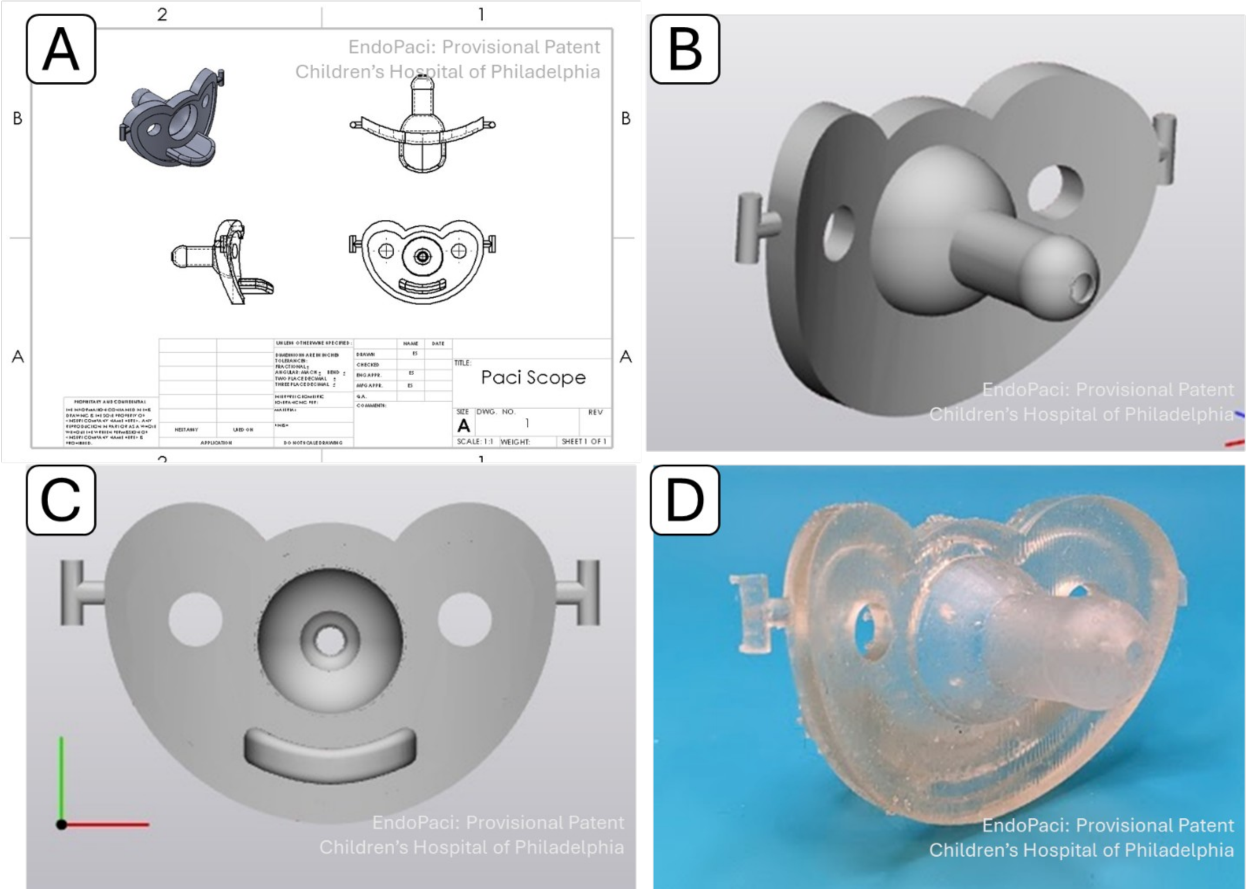
A
EndoPaci: Provisional Patent
Children's Hospital of Philadelphia
2
1
B
A
UNLESS OTHERWISE SPECIFIED:
NAME
DATE
DRAWN
CHECKED
ENG APPR.
MFG APPR.
Q.A.
COMMENTS:
TITLE:
Paci Scope
SIZE
DWG. NO.
REV
A
1
SCALE: 1:1
WEIGHT:
SHEET 1 OF 1
PROPRIETARY AND CONFIDENTIAL
NEXT ASSY
USED ON
APPLICATION
DO NOT SCALE DRAWING
B
EndoPaci: Provisional Patent
Children's Hospital of Philadelphia
C
EndoPaci: Provisional Patent
Children's Hospital of Philadelphia
D
EndoPaci: Provisional Patent
Children's Hospital of Philadelphia
