# Supplementary material for: Anesthesia‐free transoral endoscopy using a novel modified pacifier in early infancy
Source: J Pediatr Gastroenterol Nutr. 2026 Jun 5;83(2):331–4. doi: 10.1002/jpn3.70463 (PMC13446647; doi:10.1002/jpn3.70463)
Supplement: Supplementary file 1 [file JPN3-83-331-s001.docx]

| **Supplemental Table 1. Experience and Perceptions of Transpacifier Endoscopy (TPE) Among Key Stakeholders.** | | | | | |
| --- | --- | --- | --- | --- | --- |
| **Question** | **1** | **2** | **3** | **4** | **5** |
| **Parent and Caregiver Perspectives (n = 6)** | | | | | |
| I understand the benefits of doing the endoscopy without anesthesia | 0 | 0 | 0 | 0 | 6 (100%) |
| I was nervous about my child having the procedure done in a new instead of the usual way | 1 (16.7%) | 2 (33.3%) | 1 (16.7%) | 2 (33.3%) | 0 |
| I am glad that my child had the endoscopy through the pacifier | 0 | 0 | 0 | 0 | 6 (100%) |
| I was worried that my child would feel pain during the TPE | 0 | 2 (33.3%) | 1 (16.7%) | 2 (33.3%) | 1 (16.7%) |
| If my child must have this procedure again, I prefer that it be done this way | 1 (16.7%) | 0 | 0 | 0 | 5 (83.3%) |
| **Pediatric Gastroenterologist Feedback (n = 24)** | | | | | |
| I am aware that anesthesia during the first year of life can be harmful to brain development | 0 | 0 | 5 (17.9%) | 16 (57.1%) | 7 (25.0%) |
| I think that performing endoscopy on infants via a pacifier is a good idea | 0 | 1 (3.6%) | 9 (32.1%) | 11 (39.3%) | 7 (25.0%) |
| I plan to use the TPE approach, if it is shown to be comfortable for the patient | 0 | 0 | 6 (21.4%) | 13 (46.4%) | 9 (32.1%) |
| Being able to perform endoscopy on infants outside of the operating room is a major advantage | 0 | 2 (7.1%) | 2 (7.1%) | 11 (39.3%) | 13 (46.4%) |
| I think unsedated endoscopy is the future of upper gastrointestinal endoscopy for infants | 0 | 4 (14.3%) | 13 (46.4%) | 5 (17.9%) | 6 (21.4%) |
| I want to learn how to perform TPE | 2 (7.1%) | 3 (10.7%) | 6 (21.4%) | 11 (39.3%) | 6 (21.4%) |
| **Definitions:** Survey responses were scored on a 5-point Likert scale: 1, strongly disagree; 2, disagree; 3, no opinion; 4, agree; 5, strongly agree. | | | | | |
| *Note:* Adapted from Scherer C, Sosensky P, Schulman-Green D, et al. Pediatric Patients' and Parents' Perspectives of Unsedated Transnasal Endoscopy in Eosinophilic Esophagitis: A Qualitative Descriptive Study. J Pediatr Gastroenterol Nutr 2021;72(4):558-62. | | | | | |
